# Arterial Stiffness in Type 1 Diabetes: The Case for the Arterial Wall Itself as a Target Organ

**DOI:** 10.3390/jcm10163616

**Published:** 2021-08-16

**Authors:** José-Miguel González-Clemente, Albert Cano, Lara Albert, Olga Giménez-Palop, Ana Romero, Eugenio Berlanga, Joan Vendrell, Gemma Llauradó

**Affiliations:** 1 Department of Endocrinology and Nutrition, Hospital Universitari Parc Taulí, Institut d’Investigació i Innovació Parc Taulí I3PT, Universitat Autònoma de Barcelona, 08208 Sabadell, Spain; ACANO@tauli.cat (A.C.); lalb85@gmail.com (L.A.); ogimenez@tauli.cat (O.G.-P.); aromerogr@tauli.cat (A.R.); 2 Centro de Investigación Biomédica en Red de Diabetes y Enfermedades Metabólicas Asociadas (CIBERDEM), Department of Endocrinology and Nutrition, Instituto de Salud Carlos III, 28029 Madrid, Spain; jvortega@gmail.com (J.V.); gllauradoc@gmail.com (G.L.); 3 Clinical Laboratory, Biochemistry Department, UDIAT, Institut d’Investigació i Innovació Parc Taulí I3PT, Universitat Autònoma de Barcelona, 08208 Sabadell, Spain; eberlanga@tauli.cat; 4 Department of Endocrinology and Nutrition, Hospital Universitari Joan XXIII de Tarragona, Institut d’Investigacions Sanitàries Pere Virgili (IISPV), Universitat Rovira i Virgili, 43005 Tarragona, Spain; 5 Department of Endocrinology and Nutrition, Institut Hospital del Mar d’Investigacions, Mèdiques (IMIM), Universitat Autònoma de Barcelona, 08003 Barcelona, Spain

**Keywords:** arterial stiffness, pulse wave velocity, cardiovascular risk, microvascular complications, type 1 diabetes, lipoprotein particles, insulin resistance, double diabetes

## Abstract

Arterial stiffness (AS) integrates the cumulative burden of known and unknown cardiovascular risk factors on the elastic wall of large arteries along the lifespan of an individual. As a marker of vascular aging, AS is an independent predictor of cardiovascular events and improves cardiovascular risk prediction when added to the Framingham Risk Score. In addition, AS may affect the microvasculature and promote the development of microvascular complications. Its impact on both the macro- and microvasculature has led to the concept that the arterial wall itself should be considered as a target organ. Here, we review the biological and clinical consequences of AS on the macro- and microvasculature and the measurement of AS in routine clinical practice. We also discuss the pathophysiological mechanisms underpinning AS development using diabetes and, in particular, type 1 diabetes, as a disease model with a high risk of cardiovascular events and microvascular complications that are accelerated by AS.

## 1. Introduction

Cardiovascular (CV) disease is the leading cause of global mortality and a major contributor to disability [1]. Predicting CV events in the general population is commonly achieved by using one of several classical risk prediction tools that consider both unmodifiable (e.g., age, sex) and modifiable (e.g., cholesterol, blood pressure) risk factors, for example, the Framingham Risk Score [2] or the European Systematic COronary Risk Evaluation (SCORE) [3]. As patients with diabetes are at very high CV risk, specific scores for predicting CV events are also available for both type 1 (T1D) [4] and type 2 (T2D) diabetes [5]. Nonetheless, how well these scores perform in daily clinical practice is unclear [6], and the use of other CV biomarkers has been proposed to improve upon CV risk predictions. One such biomarker is arterial stiffness (AS), which is considered as the central paradigm of vascular aging [7]. AS integrates the cumulative harm of both known and unknown CV risk factors on the wall of large elastic arteries (chiefly, the aorta), along the entire life (even pre-birth) of an individual. Consequently, it is not surprising that AS (measured as aortic pulse velocity (aPWV)) is an independent predictor of CV events in the general population and improves CV risk prediction when added to the Framingham Risk Score [8]. There is accumulating evidence supporting the concept that AS underpins the development of complications at the microvasculature level [9,10]. Against this background, the present review will focus on: (1) the biological and clinical consequences of AS on both macro- and microvasculature; (2) the measurement of AS in daily clinical practice; (3) the pathophysiological mechanisms implicated in AS development; (4) AS in the context of T1D and; (5) future directions. While it is known that AS may affect almost (if not all) all chronic conditions, some of them among the most prevalent worldwide (e.g., obesity, hypertension) [11,12], this review will focus on T1D because of its high risk of both CV events and microvascular complications.

## 2. Pathophysiological and Clinical Consequences of Arterial Stiffness

**Figure 1:**
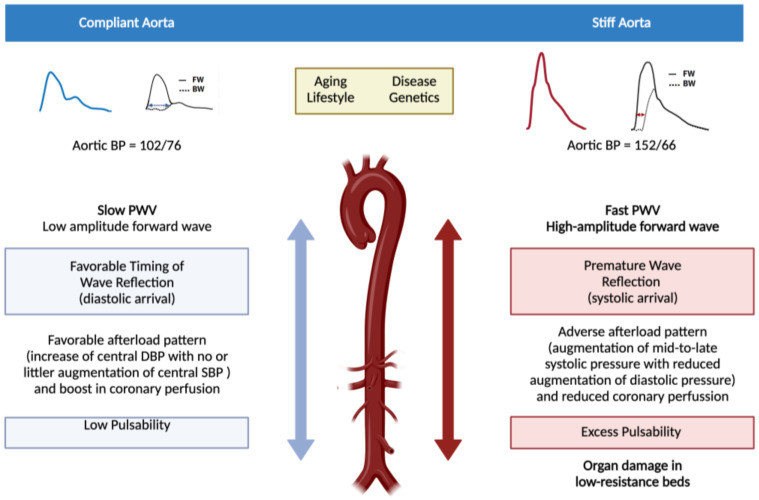
Impact of arterial stiffness on both the macro- and microvasculature. The figure compares a compliant aorta with a stiff aorta. In the left side a compliant (elastic) aorta is represented while the right represents a stiff aorta (vascular aging). Pulse waveform with their respective components of forward wave (FW, left ventricle to peripheral) and backward or reflected wave (BW, peripheral circulation to left ventricle) in both situations are shown. When the aorta is compliant, both components of the pulse wave (forward and backward) are slow and the backward component reaches the left ventricle in diastole with no increase in the physiological aortic pulse pressure and favoring myocardial perfusion (which mainly occurs in diastole). Under these conditions, blood flow reaches the microcirculation of the organs with low resistance and high flow with low pulsability and energy (left side of the figure). When the aorta stiffens, pulse wave velocity increases in both of its components, the forward component increasing the systolic blood pressure and the afterload of the left ventricle (first step towards its hypertrophy and failure) whereas the backward component reaches the left ventricle in the mid-to-late systole, compromising myocardial perfusion. In addition, blood flow increases its pulsability and energy when arriving at the low resistance high flow target organs, thus promoting their damage (right side of the figure).

**Figure 2:**
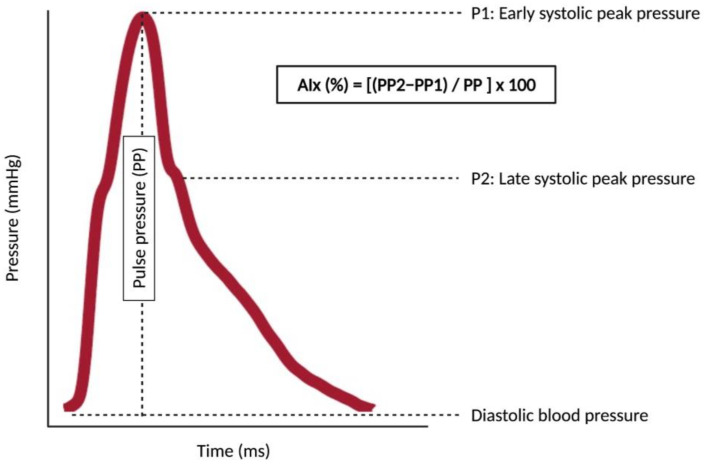
The concept of augmentation index and augmentation pressure. The figure shows a pulse waveform with two peaks: P1 is an early systolic peak pressure (derived from the forward component of the pulse wave) whereas P2 is a late systolic peak pressure (derived from the backward component of the pulse wave). Pulse pressure (PP) is the difference between the central systolic blood pressure and the central diastolic blood pressure. PP2 is the pulse pressure of the P2, and PP1 is the pulse pressure of the P1. The augmentation pressure is the difference between the PP2 and the PP1. The augmentation index (AIx) is the augmentation pressure divided by the PP and multiplied by 100 to give as a percentage. When the aorta stiffens, reflected waves arrive earlier in the systole, the P2 value is higher as well as the augmentation pressure and the AIx. As the AIx is highly dependent on heart rate, it should be reported as its value for a heart rate of 75 bpm (AIx75). The assessment of AIx75 requires only one recording site (usually radial or carotid) for recording the pulse waveform with a device provided with a transfer function for calculating the AIx as well as other pulse waveform-derived measures (e.g., subendocardial viability ratio), which makes its assessment easier than the evaluation of pulse wave velocity (see Section 3).

The presence of AS in large elastic arteries (e.g., aorta) may promote the development of both macro- and microvascular complications through hemodynamic mechanisms [9,13,14]. Beyond its conduit function, the elasticity of the aorta under physiological conditions “cushions” the changes in blood pressure caused by the intermittent left ventricle (LV) ejections. This cushioning capacity maintains central systolic blood pressure (SBP) low while avoiding the excessive fall in central diastolic blood pressure (DBP) in the forward component of the pulse wave (from LV to peripheral circulation). As coronary arteries receive blood mainly during diastole, this cushioning (or stretching) effect allows for proper perfusion of the myocardium. In the microvasculature, cushioning protects against potentially harmful fluctuations in blood pressure and flow, especially in organs with high flow and low resistance such as the kidney, the retina or the brain [9]. AS of the aorta diminishes its elasticity and the cushioning effect is lost, thus increasing SBP (which may cause systolic hypertension) and afterload in the LV as a first step towards hypertrophy, remodeling, dysfunction and, ultimately, failure. The loss of the cushioning function of the aorta concomitantly reduces DBP and, consequently, coronary perfusion, promoting myocardial ischemia. Additionally, loss of cushioning at the aortic level produces barotrauma (due to an increase in pulsatile pressure) and excessive shear stress (due to an increase in pulsatile flow) in the microvasculature, the so-called “tsunami effect” [15]. This reduces capillary transit time and metabolic exchange, leading to organ damage [13]. The loss of elasticity of the aorta due to AS also affects the backward or reflected component of pulse waves (from peripheral circulation to LV). Under physiological conditions, pulse waves generated in the LV and transmitted along the arterial wall (the forward component of the pulse wave) are partially reflected at countless sites along the arterial tree (e.g., points of branching). These reflected waves (the backward component of the pulse wave) are transmitted back to the proximal aorta where they merge with the next forward component of the pulse wave from the LV, a phenomenon that can be detected using pulse waveform analysis (PWA) to assess the augmentation index (AIx). Normally, the reflected waves reach the aorta predominantly in diastole, increasing central DBP and favoring coronary perfusion. However, under conditions of AS, pulse wave velocity (both forward and backward) increases and reflected waves reach the proximal aorta earlier, in mid-to-late systole, thus augmenting central SBP and reducing central DBP and coronary perfusion (an effect that can be assessed using the subendocardial viability ratio (SEVR) in the PWA) [15]. SEVR can be calculated as previously described [16]. Figure 1 represents graphically the impact of AS on both macro- and microvasculature and Figure 2 represents the concept of Aix and augmentation pressure.

It is known that aPWV is associated with carotid atherosclerotic (echogenic) plaques, independently of classical CV risk factors [17]; however, AS (or arteriosclerosis) should not be confused with arterial wall atherosclerosis [18]. The relationship between AS and atherosclerosis is bi-directional, with AS initiating a vicious cycle that may promote clinical CV events. Arterial wall stiffening is not uniform along the arterial tree, and some areas are more affected than others. In these latter areas the shear stress is higher, which favors the accumulation of circulating low-density lipoprotein (LDL) cholesterol to initiate the formation of the atherosclerotic plaque in the innermost layer of arterial wall—the intima (see below) [19]. Additionally, greater arterial wall stiffness favors the rupture of “established” atherosclerotic plaques and the development of clinical CV events [20]. Finally, the accumulation of cholesterol itself in the arterial wall may induce AS through several mechanisms, such as inflammation and endothelial dysfunction (see below) [21].

A recent excellent review has summarized the potential clinical consequences of AS on the microvasculature [9]. Briefly, AS may be involved in the development of several dysfunctions in the brain (e.g., cognitive impairment, dementia), the heart (e.g., myocardial dysfunction, heart failure, ischemia), the kidney (chronic kidney disease) and the liver (e.g., insulin resistance, non-alcoholic steatohepatitis), among other potential target organs with high-flow low-resistance.

## 3. Measuring Aortic Stiffness in Daily Clinical Practice

Consensus documents from European [22] and American [8] associations have established that the gold standard method for measuring AS is aPWV, assessed as carotid-femoral PWV (cfPWV). Indeed, the predictive value of cfPWV for CV events was found to be largely superior to carotid-brachial PWV, AIx and central pulse pressure in the Framingham Heart Risk Study [8]. In addition, international age-specific normal and reference standards of AS have been established using cfPWV data from over 16,000 individuals [23].

cfPWV (measured in m/s) is calculated by dividing the traveled distance of the pulse wave between the common carotid and the common femoral recording sites by its transit time between these two sites (velocity = distance/time), with the highest cfPWV equating to the greatest AS. Although the relationship between cfPWV and CV risk is continuous, a value >10 m/s for cfPWV has been proposed as a cut-off score for clinical concern [24]. In the aforementioned study in over 16,000 individuals [23], indicative 90th percentile age-specific values for cfPWV in Europeans with optimal or normal blood pressure and no other CV risk factors were reported as: <30 years, 7.1 m/s; 30–39 years, 8 m/s; 40–49 years, 8.6 m/s; 50–59 years, 10 m/s; 60–69 years, 13.1 m/s and ≥70 years, 14.6 m/s [23]. These values can be used for estimating the biological age (vascular age) of any given individual.

Although invasive methods are available for measuring cfPWV (with pressure catheter recordings), they are complex, costly and raise ethical issues. Consequently, they are usually used in validation studies of non-invasive methods. Non-invasive methods have been classified into two categories: (1) direct methods, and (2) indirect methods [25,26,27]. Direct methods use two probes and/or cuffs for recording, whereas indirect methods only use one. Direct methods include magnetic resonance imaging of the aorta, echocardiography, applanation tonometry and oscillometry [27,28]. Magnetic resonance imaging is the best technique for assessing distance, but it does not have sufficient resolution for the precise measurement of transit time. In addition, it is a complex and expensive technique, and no large studies have been reported in the general population with this technique. The other three direct non-invasive techniques exploit the superficial and relatively close location of the common carotid and the common femoral arteries to the aorta for detecting signals from the pulse wave (pressure, distension or flow) with sufficient temporal resolution for calculating the cfPWV. Applanation tonometry is the most extensively used non-invasive technique for evaluating cfPWV [29], and can be performed with several commercially-available systems, such as the Sphygmocor^®^, (AtCor Medical, Sidney, Australia) which was utilized for the aforementioned international age-specific normal/reference standards of AS [23]. This method is accurate for assessing the transit time but does not provide a direct measurement of the distance between the common carotid and the common femoral sites. To measure distance, one option is subtracting from the distance between the femoral site and the suprasternal notch, the distance between the carotid site and the suprasternal notch. However, the current consensus is to consider the distance between the carotid and the femoral sites as 80% of the distance measured using a tape meter, because international reference standards of cfPWV have been reported using this methodology [9,23]. These standards include equations for transforming distances according to the “subtracting method” into distances obtained with the “80% method” [23]. In any case, measuring cfPWV is a proxy of aPWV, as it does not consider the pulse wave traveling in the ascending aorta and in the proximal part of the aortic arch, which are the most elastic parts of the aorta and contribute most to its cushioning function. In addition to cfPWV measurements, other devices have been developed for recording signals at more distal sites, using cuffs at the brachial artery and ankle or fingers/toes. These devices obtain measures such as brachial-ankle PWV or finger-toe PWV. The brachial-ankle PWV has been evaluated mostly in Asian populations and it has been demonstrated to be predictive of CV risk, but with lower correlation and agreement with invasively determined aPWV than using cfPWV [25].

Although manufacturers of some devices claim to be able to assess PWV with a single brachial cuff pressure recording (indirect methods, e.g., Mobil-O-Graph^®^,Industrielle Entwicklung Medizintechnik, Stolberg Germany; Arteriograph^®^, TensioMed, Budapest, Hungary), it is impossible to precisely measure velocity with only one site of recording. These methods can only provide some estimation of PWV using specific mathematical models and algorithms [25]. For example, the estimations provided by Mobil-O-Graph^®^, a 24-h ambulatory central blood pressure system, are 99% explained by age and SBP (but this percentage is only 40% when cfPWV is measured with direct methods), and have not been properly validated against invasively-measured aPWV [25].

In addition to the above considerations regarding the method to assess AS, it is important to follow existing recommendations when measuring AS in clinical practice. These recommendations are listed in Table 1.

## 4. Pathophysiological Mechanisms Implicated in Stiffening of Large Elastic Arteries

**Figure 3:**
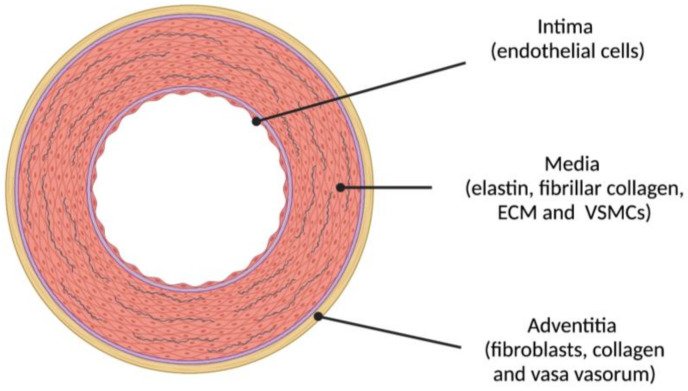
The three layers of the elastic arterial wall are: (1) intima (endothelial cells); (2) media (concentric layers of elastin, fibrillar collagen and other proteins integrating the extracellular matrix (ECM) and vascular smooth muscle cells (VSMCs)); and (3) adventitia (fibroblasts, collagen and, in larger arteries, vasa vasorum). Atherosclerosis initiates in the intima whereas arteriosclerosis (AS) mostly affects the media.

The arterial wall is the target of AS and is made up of three different structural layers separated by elastin fibers arranged in fenestrated sheets: the intima, the media and the adventitia that are schematically represented in Figure 3.

Several pathophysiological mechanisms act on this anatomical substrate and participate in the development of AS through processes that are still not completely understood. Here we will only provide a brief overview. The media layer of the arterial wall is the layer most affected by AS: the ECM supports mechanical loads (elastin fibers provide elasticity while collagen fibers provide rigidity) and VSMCs regulate vascular tone (actin-myosin contraction) and participate in the mechanotransduction of signals in the arterial wall. There are two important elements to be considered in the scenario of AS. The first is the fragmentation of elastin and its substitution for collagen fibers, which stiffens the arterial wall. Fragmentation occurs due to mechanical fatigue and/or elastin proteolysis. Elastin has an extremely long half-life (mean of 74 years) and has minimal turnover [10]. Elastin is deposited in the arterial wall during fetal life and infancy and its fragmentation is virtually irreparable. The mechanical stress of 30 million cardiac ejections that the arterial wall must cushion every year is a major cause of elastin fragmentation. In addition, elastin fibers are very sensitive to proteolysis from different elastases that are secreted by pro-inflammatory cells in the arterial wall (e.g., metalloproteinases), especially if they have been modified by glycation, peroxidation or other post-translational modifications beforehand [9]. Collagen deposits at sites of elastin degradation increase the rigidity of the arterial wall. In contrast to what occurs with elastin fibers, advanced-glycation end-products of proteins, mediated by exposure to glucose, increase the resistance of collagen to proteolysis. Interaction of advanced-glycation end-products with their receptors further promotes stiffening of the arterial wall, enhancing inflammation, oxidative stress and calcification [9]. The second element is VSMCs, which are known to have a critical role in the development of AS and, consequently, are under intensive investigation as potential therapeutic targets for preventing or reversing AS [31]. VSMCs are sensitive to several factors including mechanotransduction signals, oxidative stress, metabolic signals and genetic and epigenetic factors that, overall, determine their phenotype [32]. VSMCs primarily regulate vascular tone and provide structural integrity to the vascular tree (contractile phenotype). When stressed, VSMCs undergo a phenotypic switch through dedifferentiation and become hyperproliferative, migratory and/or proinflammatory (proliferative phenotype). VSMCs can assume a macrophage-like phenotype in developing atherosclerotic plaques, secrete ECM proteins such as collagen, inducing fibrosis, or become osteogenic and promote medial calcification [33]. Nitric oxide reduction in endothelial cells (endothelial dysfunction), as a result of shear stress on the arterial wall, may also promote a proliferative phenotype of VSMCs. Indeed, the primary molecular mechanisms of AS are oxidative stress and low-grade inflammation [34]. In the specific case of diabetes, both hyperglycemia and insulin resistance are likely involved in the development of AS [32]. Results from the Atherosclerosis Risk in the Communities epidemiological study suggest that hyperglycemia contributes to AS development beyond its effects in promoting the atherosclerosis of the arterial wall [35]. Chronic hyperglycemia not only induces the production of advanced-glycation end-products and collagen cross-linking, but also promotes the proliferative VSMC phenotype (with an activation of their angiotensin II receptors) and stimulation of matrix metalloproteinases 2 and 9. Insulin resistance is associated with an increase in collagen synthesis and an increase of the expression of a number of pro-inflammatory genes. In addition, the activation of the local (VSMCs) renin-angiotensin-aldosterone system in response to hyperglycemia and sodium retention seems to play a critical role in the development of fibrosis, oxidative stress, inflammation, endothelial dysfunction and vascular remodeling [36].

The development of AS is also influenced by genetic and epigenetic factors, although a comprehensive analysis of these factors is beyond the scope of this review. Briefly, the heritability of AS itself is moderate [10,32]. Few common genetic variants have been associated with measures of AS, and it is much more common to find associations with mechanisms involved in AS development, such as inflammation or lipid disturbances. For example, a single nucleotide polymorphism in the apolipoprotein A5 gene has been associated with higher AS (higher brachial-ankle PWV) and lower adiponectin levels in Koreans with low levels of high-density lipoprotein (HDL) [37]. In contrast to genetic factors, epigenetic regulators are thought to play a critical role in the VSMC phenotypic switch, as a causal mechanism in the development of AS. One of these epigenetic regulators is made up by noncoding RNAs, particularly microRNAs. Indeed, a recent review identified more than 50 microRNAs in experimental models as potential candidates for the development of AS [38]. However, to the best of our knowledge, only one study in humans has identified an association between cfPWV and the circulating levels of microRNAs. In this study, lower levels of microRNA-21 were associated with lower cfPWV [39]. Of note microRNA-21 together with microRNA-155 and microRNA-146a are considered biomarkers of the aging process [40]. In addition to microRNAs, sirtuins have been classically involved in the pathogenesis of AS. Sirtuin-1, a class III protein lysine deacetylase, protects against AS in experimental models, likely mediated by its anti-inflammatory and antioxidant effects. Likewise, the promoting effect of smoking on AS seems to be explained by the inhibitory effect of nicotine on sirtuin-1 [32].

## 5. Arterial Stiffness in Diabetes Mellitus. The Case of Type 1 Diabetes Mellitus in Adults

Studies on AS in diabetes have been done in adolescents, young adults and adults. Studies in adolescents and young support the concept that an increase in AS can be detected early in the course of the natural history of diabetes. In fact, an increase in cfPWV has been detected in adolescents and young adults with either T1D or T2D [41]. In addition, this increase is more prevalent than nephropathy, retinopathy and peripheral neuropathy after a mean duration of diabetes of 7.9 years in both T1D and T2D (T1D: 11.6% vs. 5.8%, 5.6% and 8.5%, respectively; T2D: 47.4% vs. 19.9%, 9.1% and 15.7%, respectively) [42]. These results are in accordance with the hypothesis that an increase in AS may be involved in the development of microvascular complications of diabetes.

The current review will be focused on studies performed in adults with T1D. Accumulating evidence suggests that adults with T1D suffer from an accelerated aging process and, in particular from an accelerated vascular aging. Patients with T1D at age 20 face a loss in life-expectancy of 11 (men) to 13 (women) years, which is approximately 2 years per decade of T1D duration, with CV disease being the most common cause of death [43]. Indeed, the absolute CV risk in men and women with T1D aged 45–55 years is similar to that of men in the general population 10–15 years older [44]. Accordingly, people with T1D show an increase in brachial pulse pressure (a crude surrogate of aortic AS) up to 15–20 years earlier than in healthy controls [45]. In addition, a number of studies in the last decades have addressed the issue of AS (vascular aging) in patients with T1D, with most studies using AIx as a surrogate of aortic AS. In 2012, our group was the first to report an increase in AS, as assessed by cfPWV, in patients with T1D as compared with healthy controls [46]. Table 2 summarizes studies in adults with T1D that have addressed the potential association between surrogates of AS and clinical manifestations of both macro- and microvascular complications, total mortality, and CV mortality.

These studies reveal that several surrogates of AS are associated with both macro- and microvascular complications in T1D. Of note, higher cfPWV has been associated with an increase in CV disease, retinopathy and neuropathy, and cardiac autonomic neuropathy in a cohort of patients with T1D from the Steno Diabetes Center [52], supporting the concept that the arterial wall should be viewed as a target organ. Our group recently reported two cut-off points of cfPWV (using the subtracting method) for identifying patients with T1D (and no previous CV disease) with moderate/high risk (>10%) and high risk (>20%) for CV events along a 10-year period according to the scores obtained with the Steno Type 1 Risk Engine [44]. This score requires 10 clinical variables, and we proposed to simplify the assessment of CV risk by evaluating of cfPWV, with the caveat that further evaluation will be needed in appropriate prospective cohorts. More recently, higher levels of AIx have been shown to be predictive of all-cause mortality and a combination of CV and/or diabetes-related mortality [56].

In addition to these studies, others have investigated potential biomarkers and/or therapeutic targets for preventing/reversing AS in T1D. For instance, our group reported an association between higher cfPWV levels and higher levels of low-grade inflammation [46], which accords with our previous data using brachial pulse pressure as a surrogate of AS [57]. Our group also reported a positive correlation between cfPWV and the accumulation of serum and skin auto-fluorescent advanced-glycation end-products [58]. With regards to molecules potentially involved in arterial wall calcification, we and others have reported an association between higher AS (cfPWV) and higher circulating levels of FGF-23 and lower levels of circulating vitamin D [59], as well as higher levels of circulating parathyroid hormone [60]. Our group also recently performed a nuclear magnetic resonance study in patients with T1D and no previous CV disease, and found that cfPWV correlates positively with the total number of VLDL particles and subclasses and their content in triglycerides, and negatively with LDL and HDL particle sizes, although they had a better conventional lipid profile than healthy controls [61]. These findings support a potential role of insulin resistance in the development of AS in T1D, in the context of what has been termed double diabetes [62].

## 6. Arterial Stiffness in Diabetes: Future Directions

It is generally thought that it is not yet the time for assessing cfPWV in clinical practice, in part for the complexity of the technique and for the lack of experience in its implementation. Currently, no clinical guideline recommends the routine assessment of AS (cfPWV) in daily clinical practice, although some evidence suggest that its determination could be helpful for a better classification of individuals at moderate or intermediate CV risk [9,63,64]. In fact, a recent position paper on the usefulness of several biomarkers for primary and secondary cardiovascular prevention issued by the European Society of Cariology has stated that the methods for assessing cfPWV are easier to use and more standardized than the ultrasonographic methods for assessing atherosclerosis in the carotid artery (B-mode) [65]. In addition, cfPWV is more sensitive to changes, more useful for guiding pharmacological treatments and even better for reclassifying patients than these carotid ultrasonographic techniques [65].

Recently a number of research gaps to be investigated in the field of AS has been identified, including the lack of well-designed clinical trials with cfPWV as a primary endpoint along with major CV events [10,14]. Nonetheless, as AS represents the biological age of any given individual, and because patients with diabetes always have a loss in their life-expectancy (that is a biological age > chronological age), we propose that the periodic assessment of cfPWV could be included in the standard of care of patients with diabetes. While we acknowledge that there is a paucity of appropriate clinical studies to support this proposition, it should be remembered that the major cause of mortality in the general population is advanced (biological) age, and that assessing biological age could be of great interest in patients with diabetes for advancing precision medicine approaches. Two additional observations support this proposal. First, cfPWV is a simple, inexpensive and non-invasive marker that can be monitored across the entire life of a given individual. In fact, the current assessment of cfPWV by applanation tonometry could be simpler than performing a routine funduscopic examination or searching for atherosclerotic plaques in the carotid by echography. Second, we know that some interventions may reduce cfPWV. Non-pharmacological interventions such as weight-loss by diet or exercise training may decrease AS [29]. Medications for hypertension such as inhibitors of the renin-angiotensin system or calcium channel blockers may also decrease cfPWV, while beta-blockers and diuretics would have minimal impact, if any, on AS [29]. Statins and ezetimibe may also decrease cfPWV, although the effect of new anti-PCSK9 drugs (alirocumab, evolocumab) could have a much more important decreasing effect on cfPWV [66]. Regarding diabetes, empagliflozin and liraglutide, which have demonstrated CV benefits in T2D in their respective CV outcome trials, have recently been demonstrated to reduce cfPWV over a 1-year period and that this effect is stronger in combination than with either agent used alone [67].

## 7. Conclusions

AS integrates the entire harm on the arterial wall of large arteries along the whole life of a given individual. It is considered a sign of vascular aging and a measure of biological age. The gold standard for measuring AS is the assessment of cfPWV, which can be typically measured in clinical practice by applanation tonometry. cfPWV has been demonstrated to improve the CV risk prediction when combined with the Framingham Risk Score. Accumulating evidence supports the concept that the arterial wall of large arteries should itself be considered as a target organ, because wall stiffening would underpin the development of macro- and microvascular complications, especially in patients with diabetes but also in most (if not all) any other chronic condition. The media layer of large arterial walls plays a critical role in stiffening, driven mainly by elastin fiber fragmentation and dedifferentiation of VSMCs. A number of pathophysiological mechanisms have been implicated in the development of AS, including low-grade inflammation, endothelial dysfunction, advanced-glycation end-product accumulation and calcification of the media layer. Biomarkers of most of these mechanisms have been identified in adults with T1D, where AS predicts total mortality and a combination of CV mortality and/or diabetes-related mortality, and is associated with microvascular complications. We propose that the periodic assessment of cfPWV in patients with diabetes should be initiated as a means for assessing their biological age and progressing towards precision medicine. However, we acknowledge that further research is needed before implementing such proposals in daily clinical practice.

## Figures and Tables

**Table 1:** Recommendations for measuring cfPWV in daily clinical practice [24,30].

Recommendations for Measuring cfPWV
1. Place: a quiet room with a stable room temperature (e.g., 21–23 °C)
2. Position: supine after at least 10 min rest
3. Measurements preferably done at the right common carotid and common femoral arteries
4. Repeated measures should be done at the same time of the day
5. No food, caffeine or smoking within 3 h before measurements
6. No speaking or sleeping during measurements
7. Data should be mean of registrations during one respiratory cycle (about 5–6 s)
8. Be aware of possible white-coat effects
9. Measure distance in a straight line (carotid-femoral). If not possible with a tape meter (e.g., obesity) an infantometer may be helpful used upside-down
10. Take mean of at least two measurements; if difference between them is >0.5 m/s perform a third measurement and take the median (not mean) of the three measurements
11. Circumstances in which measurements of cfPWV should not be performed: arrhythmia, unstable clinical situation, high-grade stenosis of carotid artery, carotid sinus syndrome.

Abbreviations: cfPWV: carotid-femoral Pulse Wave Velocity.

**Table 2:** Main studies on arterial stiffness in adults with T1D.

Author, Year, Design	Surrogate of AS	Results
Prince, 2010, cross-sectional [47]	AIx, AP, SEVR	Cardiac autonomic neuropathy associates with higher AIx and AP and lower SEVR
Prince, 2010, cross-sectional [48]	AIx, AP, SEVR	Higher AP associates with prevalent coronary artery disease. Lower SEVR associates with lower ankle-brachial index.
Gordin, 2011, prospective [49]	Brachial PP	Higher brachial PP predicts CV events but not nephropathy.
Llauradó, 2012, cross-sectional [46]	cfPWV	cfPWV is increased in T1D and associates with low-grade inflammation.
Theilade, 2012, prospective [50]	Brachial PP	Higher brachial PP predicts all-cause and CV deaths and progression to end-stage renal disease.
Gordin, 2012, cross-sectional [51]	AIx	AIx is increased in T1D and is higher in the presence of nephropathy, retinopathy and CV disease.
Theilade, 2013, cross-sectional [52]	cfPWV	Higher cfPWV associates with higher prevalence of CV, retinal, renal and cardiac autonomic diseases.
Theilade, 2014, cross-sectional [53]	AIx, AP	Higher AIx and AP associates with albuminuria and CV disease but not with retinopathy or cardiac autonomic neuropathy.
Theilade, 2014, cross-sectional/longitudinal [54]	Aortic systolic and diastolic pressures, aortic PP, SEVR	Higher aortic systolic pressure and PP, and lower aortic diastolic pressure and SEVR associates with CV disease and albuminuria. Lower SEVR predicts morality or end-stage renal disease.
Llauradó, 2019, cross-sectional [55]	cfPWV	Higher cfPWV are highly positively associated with higher CV risk according to the ST1RE.
Tynjälä, 2020, prospective [56]	AIx	Higher AIx predicts total mortality and a combination of CV and/or diabetes-related death.

Abbreviations: AS: Arterial Stiffness; AIx: Augmentation Index; AP: Augmentation Pressure; CV: cardiovascular; PP: Pulse Pressure; SEVR: Subendocardial Viability Ratio; cfPWV: Carotid-Femoral Pulse wave Velocity, ST1RE: Steno Type 1 Risk Engine; T1D: type 1 diabetes.

## Data Availability

Not applicable.
